# Three-dimensional kinematic and kinetic analysis of knee rotational stability in ACL-deficient patients during walking, running and pivoting

**DOI:** 10.1186/s40634-016-0062-4

**Published:** 2016-10-12

**Authors:** Marie Bagger Bohn, Annemette Krintel Petersen, Dennis Brandborg Nielsen, Henrik Sørensen, Martin Lind

**Affiliations:** 1 Division of Sportstrauma, Department of Orthopedics, Aarhus University Hospital, Tage Hansens Gade 2, 8000 Aarhus C, Denmark; 2 Department of Physiotherapy and Occupational Therapy, Aarhus University Hospital, Palle Juul-Jensens Boulevard 99, 8200 Aarhus N, Denmark; 3 Department of Public Health – Sport, Aarhus University, Dalgas Avenue 4, 8000 Aarhus C, Denmark

**Keywords:** ACL, ACL-deficient, Motion analysis, Stiffness, Laxity, Pivoting

## Abstract

**Background:**

Anterior cruciate ligament (ACL) deficiency leads to altered stability of the knee. The purpose of this study was to compare the dynamic, rotational stability of the knee, expressed as rotational stiffness, between anterior cruciate ligament-deficient (ACLD) knees, their contralateral intact knees (ACLI) and a knee healthy control group during walking, running and 90° pivoting. We hypothesized a larger tibial internal rotation, a smaller knee joint external moment and a lower rotational stiffness in the ACLD group compared to the ACLI and the control group.

**Methods:**

Kinematic and kinetic data were collected from both legs of 44 ACLD patients and 16 healthy controls during walking, running and a pivoting maneuver (descending a staircase and immediately pivoting 90° on the landing leg). Motion data were captured using 8 high-speed cameras and a force-plate. Reflective markers were attached to bony landmarks of the lower limb and rigid clusters on the shank and thigh (CASH model). Maximum internal tibial rotation and the corresponding rotational moment were identified for all tasks and groups and used to calculate rotational stiffness (= Δmoment /Δrotation) of the knee.

**Results:**

The tibial internal rotation of the ACLD knee was not significantly different from the ACLI knee during all three tasks. During walking and running, the tibial rotation of the control group was significantly different from both legs of the ACL-injured patient. For pivoting, no difference in tibial rotation between knees of the ACLD, ACLI and the control group was found. Knee joint external moments were not significantly different between the three groups during walking and pivoting. During running, the moments of the ACLI group were significantly higher than both the knees of the ACLD and the control group. Rotational stiffness did not differ significantly between groups in any of the three tasks.

**Conclusion:**

A high-intensity activity combining stair descent and pivoting produces similar angular rotations, knee joint external moments and rotational stiffness in ACLD knees compared to ACLI knees and the control group. During running, the ACLI knee displayed a higher external moment than the ACLD and the healthy control group. This could indicate some type of protective strategy or muscular adaptation in the ACL-injured patients.

## Background

Anterior cruciate ligament (ACL) rupture is one of the most frequent sports-related injuries in orthopedic surgery (Fu et al., 2015). Young and physically active people are prone to sustain an ACL injury, and most injuries are sustained during contact or pivoting sports. In Scandinavia, the median age for sustaining an ACL injury is 23–27 years, and the yearly incidence is 38 per 100,000 people (Granan et al., 2009). Therefore, ACL patients are often referred to as a young patient with an old knee, as long-term clinical problems such as meniscal damage and osteoarthritis (OA) development are often observed (Frobell et al., 2010, Fu et al., 2015,Lohmander et al., 2007, Risberg et al., 2016, Stergiou et al., 2007).

It is well known that an ACL deficient (ACLD) knee can exhibit pathological laxity, which often leads to complaints of knee instability from the patient (Gabriel et al., 2004, Hasegawa et al., 2015). This condition has been proposed to contribute to the development of osteoarthritis (Stergiou et al., 2007).

Functional knee assessments pre- and post-surgery often mention laxity and stability. However, from a strictly biomechanical point of view, laxity and stability are not well defined in the clinical literature. Cross defined laxity as “… the measured amplitude of joint movement within the constraints of its ligaments”, i.e. a purely kinematic measure expressed in millimeters and degrees for translational and rotational laxity, respectively (Cross 1996). Kovalski, on the other hand, defined laxity as “… the freedom of movement within a joint and is measured as joint translation at a given force load”, i.e. a kinetic measure including both the magnitude of the movement (millimeters) and the force (newton) causing the movement, bringing laxity closer to the well-defined biomechanical concept compliance (Kovaleski et al., 2008). Cross further defined instability as “a complaint from the ACL injured subjects because they lose single leg stance as the joint subluxes due to the pathological laxity”, i.e. a purely subjective measure (Cross 1996).

Because of the ambiguous definitions of laxity and the subjective definition of instability, we use the biomechanically well-defined concepts rotation, moment and stiffness in the present paper. Rotation, measured in degrees (deg), is the magnitude of the movement of the tibia about its longitudinal axis relative to the femur. Joint moment (of force), measured in newton-meters (Nm), is the magnitude of the turning force exerted by the ACL and other knee structures, equal in magnitude but opposite in direction to moments applied to the leg from the surroundings (typically the ground) causing the rotation. Stiffness, defined as change in joint moment divided by change in rotation, and hence measured in Nm/deg, is the knee’s ability to withstand moments applied to the leg from the surroundings without rotating – the stiffer the joint, the less it rotates when exposed to a certain moment. Thus, the clinical, subjective concept stability can be precisely quantified as stiffness.

Knee joint rotation and moment, and thereby stiffness, can be assessed under both static conditions and during natural movements. Static conditions are for instance with the patient reclining on the clinician’s bed or fixated in an isokinetic dynamometer (or material testing apparatus for cadaver knees), while the only restriction on assessment during natural movements is the dependency on advanced motion capture equipment, ng it takes place in a laboratory.

Static assessment has been used in several studies in both cadaver knees (Hsu et al., 2006, Kanamori et al., 2000, Yagi et al., 2002, Zantop et al., 2007) and living subjects (Louie and Mote 1987, Schmitz et al., 2008). In some of the cadaver studies, stiffness, i.e. simultaneous measurement of rotation and moment, was measured with tension in the knee joint muscles realized artificially via various pulling mechanisms, while the subjects in Louie and Mote’s study could tense and relax their muscles voluntarily. However, regardless of the type of muscle tension, it always resulted in a considerable increase in stiffness; Louie and Mote, for instance, saw stiffness increase by over 400 % when their healthy subjects went from relaxing to fully activating their knee joint muscles.

Assessment during natural movements using 3D motion analysis has by definition higher external validity compared to static assessment, both due to the movement itself, and to the subject being able to activate thee knee joint muscles in a natural way. A number of studies have reported altered transverse plane kinematics in ACLD patients during various tasks, with a possible trend toward increased internal rotation of the tibia (Andriacchi and Dyrby, 2005,Gao and Zheng, 2010, Georgoulis et al., 2003, Ristanis et al., 2005, Ristanis et al., 2006, Waite et al., 2005, Zabala et al., 2015). However, most of these studies only report kinematics, either knee range of motion (ROM) or peak knee rotation. This is problematic, since knee rotation depends on both the moment of force applied by the surroundings, causing the rotation, and on the ACL’s (and other knee joint structures’) ability to resist the rotation by creating a counteracting knee joint moment, i.e. the stiffness of these structures. Thus, in comparative studies, rotation is a valid measure of stability only when the moment is carefully measured and reported, e.g. together as stiffness. However, moments have been reported in only a few studies (Fuentes et al., 2011, Tsarouhas et al., 2010, Tsarouhas et al., 2011), and none of these calculated the actual stiffness. Interestingly, Tsarouhas et al. found that the knee joint moment of the affected side (ACL deficient or reconstructed) was constantly lower than on the unaffected side (ACL intact knee), although a similar range of tibial rotation was seen in the affected and unaffected knees in all groups (Tsarouhas et al., 2010, Tsarouhas et al., 2011). These findings indicate some kind of protective, stiffness increasing strategy in the affected knee, and, furthermore, give rise to the question if rotation can stand alone when reporting joint stability.

To our knowledge, the rotational stiffness in ACLD knees, contralateral ACL intact knees and knees of healthy control subjects during natural movements has not yet been reported. Thus, the purpose of this study was to determine knee rotational stability expressed as rotational stiffness, in ACLD and healthy knees during simulated, natural movements. Three movement tasks were analyzed: walking, running and stair descent followed by 90° pivoting. We hypothesized larger internal rotation, lower rotational moments and therefore a lower rotational stiffness in the ACLD knees compared to the ACLD subjects’ contralateral uninjured knee (ACLI) and a healthy control group (control).

## Methods

### Study design

This cross-sectional study was conducted between January 2009 and November 2010. The protocol was approved by the Region Midtjylland ethical committee (jr. nr. 20060198). Prior to participation, written informed consent was obtained from every subject.

### Subjects

Forty-four patients (18 females and 26 males) with a unilateral ACL lesion were included from a public hospital waiting list by the first author. Inclusion criteria for entering the study were: age 18–50 years, ACL injury with symptoms of instability and an uninjured contra-lateral knee. Exclusion criteria were: concomitant knee ligament injuries, previous knee ligament surgery, cartilage injuries of International Cartilage Research Society (ICRS) grade 3 or 4 and meniscus injury requiring resection of more than 50 % of a meniscus. Sixty patients were assessed for eligibility. Six of these patients were excluded prior to surgery (one declined to participate, one did not meet the inclusion criteria, and 4 were excluded for other reasons) and another ten were excluded at surgery. The control group consisted of 16 age- and sex-matched healthy subjects who had no history of lower extremity pathology or trauma.

The demographic data of the ACL injured patients and the control group is presented in Table 1. No significant differences were observed between the two groups in terms of sex, age, height, weight and body mass index (BMI). The median time since injury was 11 months (range: 2–42).

**Table 1:** Demographics of ACL injured patients and the knee healthy control group

	Patients	Control group	*P*-value
Sample size	44	16	
Sex (Female/Male)	18/26	6/10	0.12
Age (years) (mean ± SD)	25.7 ± 6.1	26.6 ± 3.6	0.94
Height (cm) (mean ± SD)	177.5 ± 9.6	178.6 ± 8.5	0.67
Weight (kg) (mean ± SD)	76.5 ± 14.9	73.7 ± 7.5	0.35
BMI (kg/m^2^) (mean ± SD)	24.1 ± 3.3	23 ± 1.5	0.08
Time since injury (months) (mean ± SD (range))	11 ± 9 (2–42)		

*SD* Standard Deviation, *BMI* Body mass index

There were 20 injured right knees and 24 injured left knees. The majority of the injuries were sports-related; 20 ruptures were sustained during soccer, 5 during skiing, 7 during handball (European team handball) and one each during field hockey, tennis, cheerleading, trampoline jumping and roller blading. One patient was injured as she was kicked directly on the knee by a horse while the remaining six patients had torsional traumas to their knee during activities of daily living.

### Testing procedure and data analysis

#### Clinical evaluation

At the time of inclusion, each patient was examined clinically and graded according to the objective IKDC grading. Passive, sagittal laxity was measured using a KT-1000 arthrometer (Medmetric® Corp., San Diego, California). Furthermore, four subjective outcome questionnaires were completed; the International Knee Documentation Committee (IKDC) subjective score (Hefti et al., 1993), the Knee Osteoarthritis Outcome Score (KOOS) (Roos et al., 1998), the Tegner score and the Lysholm score (Wright, 2009).

#### Three-dimensional motion analysis

##### Protocol

The protocol was identical for patients and control subjects and the patients were tested up to 3 weeks prior to ACL reconstruction. Hence, all participants performed three different tasks in the following order: 1) level walking, 2) running/jogging, and 3) stair descending followed by a 90° pivoting maneuver. Walking and running were performed at the participant’s self-selected speed until at least 5 successful (clean force plate contact) trials were recorded for each side and exercise. The tasks were performed on an 8 m walkway with an embedded force plate. The entire walkway was covered with a thin carpet to conceal the position of the force plate.

**Fig. 1:**
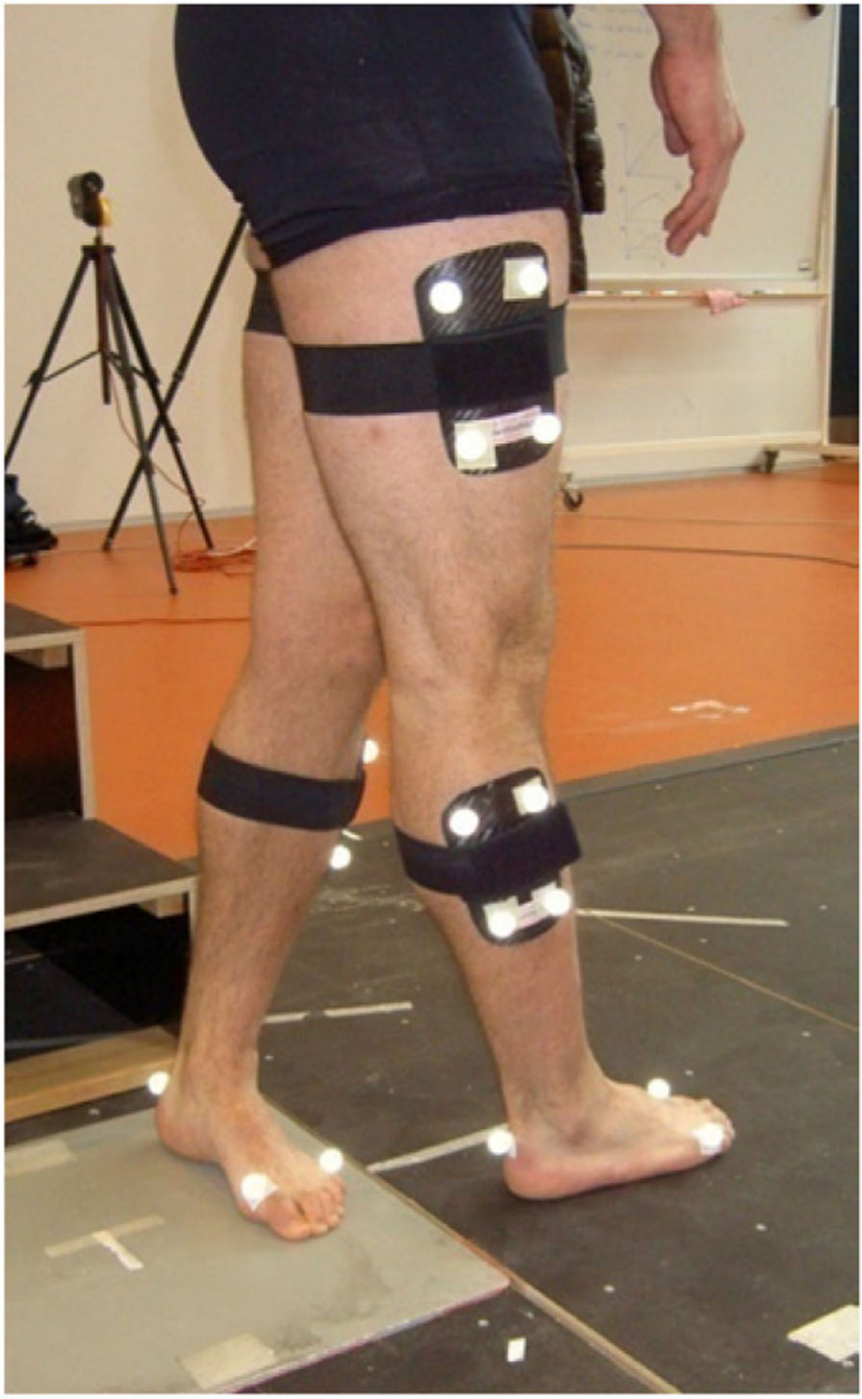
Pivoting task

Stair descending was performed on a three-step plywood staircase with no handrail, which was placed next to the force plate (Fig. 1). The staircase was constructed according to Andriacchi et al. (Andriacchi et al., 1980) (rise 21 cm, run 25 cm, width 48 cm). The participants were asked to descend the staircase at their own pace. Following contact with a force plate at the bottom of the staircase, the subjects were instructed to make a pivoting maneuver by moving the swing leg through a 90° arc across the stance leg and to contact the ground with the foot of the swing leg at a 90° angle relative to the stance foot. Full plantar side force plate contact was required. The subjects then walked a few steps away from the plate. This pivoting maneuver was designed to impart an internal rotation of the shank relative to the thigh of the stance leg (Fig. 1). Similar procedures have been described in several previous studies (Georgoulis et al., 2003, Ristanis et al., 2003, Webster et al., 2010). At least 10 successful trials for each leg of the participants were recorded. The patients always started out by pivoting on their ACLI knee followed by the ACLD knee. To ensure a constant procedure during stair descent and pivoting, each trial was carefully supervised.

##### Instrumentation for data collection

**Fig. 2:**
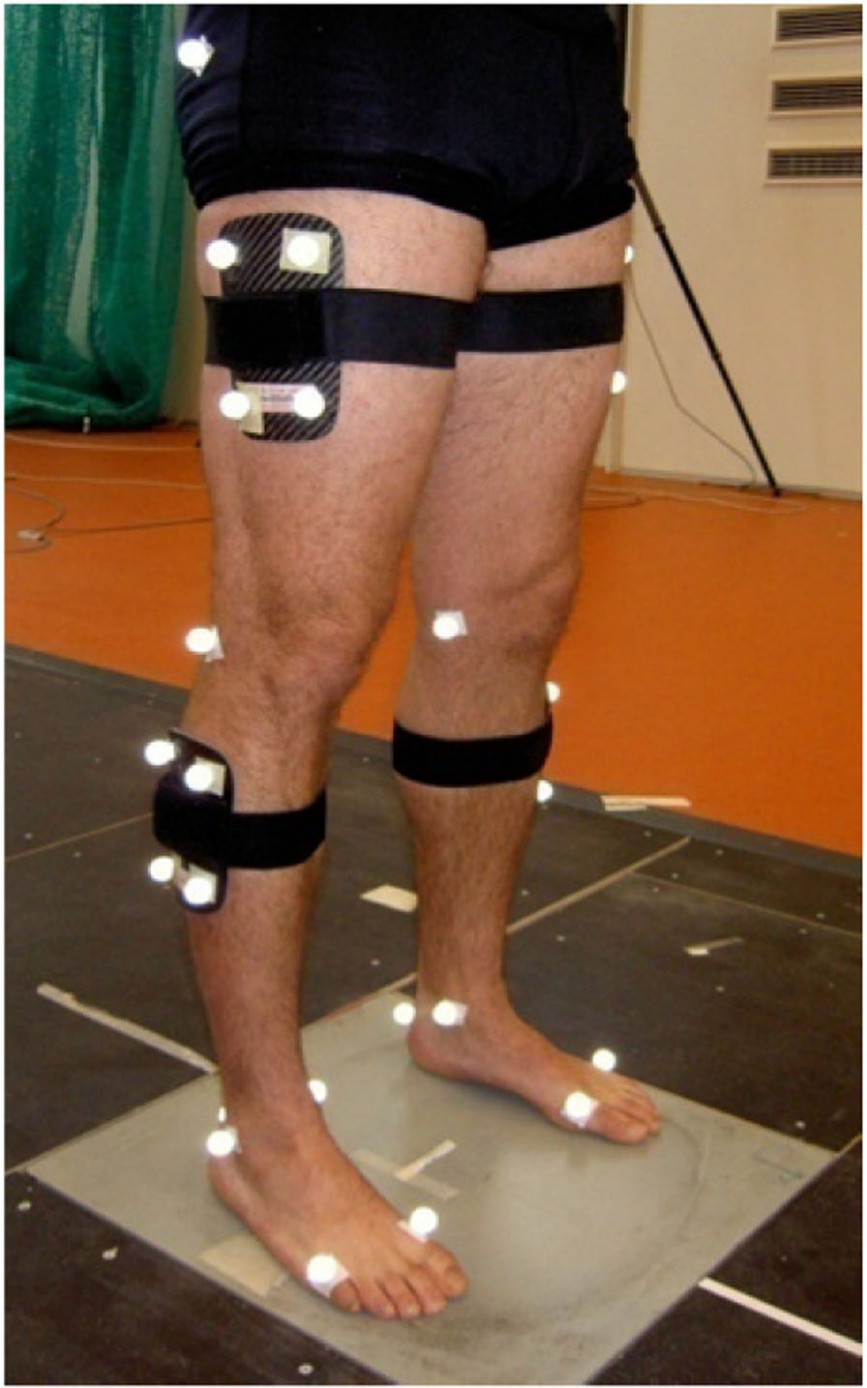
Reflective markers

All trials were performed with bare feet. Automatic tracking was facilitated by 16 reflective markers placed on anatomical landmarks and as clusters on rigid plates on each leg. Eight markers were fitted on bony prominences (greater trochanter, medial and lateral femoral condyle, medial and lateral malleolus, heel, and 1^st^ and 5^th^ metatarsal head) to define anatomical planes and joint centers, while the remaining eight markers were placed as two four-marker clusters on the shank and thigh segment, respectively (Fig. 2). All markers were placed by one investigator.

To measure the position of the reflective markers, eight optoelectronic motion capture cameras (ProReflex MCU 1000, Qualisys Medical AB, Gothenburg, Sweden) operating at 240 frames per second were used together with Qualisys Tracking Manager (QTM) software on a personal computer. The system was calibrated prior to each data collection session. To obtain a reference point for the markers, a static trial was obtained before performing the protocol with the subject in quiet standing.

The collected trajectory data were gap filled if required (gaps were rare and typically only 2–3 frames wide, in very rare occurrences up to 10 frames) in QTM using NURBS interpolation and exported to Visual3D software (C-motion Inc., Kingston, Canada) where a Visual3D Hybrid Model for ideal rigid segments and 6-degrees-of-freedom (6DOF) was applied. The marker position data were low-pass filtered using a 2^nd^ order Butterworth digital filter with one bidirectional pass (effectively making it a 4^th^ order filter) and an effective cut-off frequency of 6 Hz.

Ground reaction force (GRF) was sampled simultaneously using an AMTI OR6-6 force plate (Advanced Medical Technology Inc., MA, USA) sampled at 960 Hz. The GRF data were low-pass filtered with a cut-off frequency of 30 Hz.

##### Data analysis

From the marker positions and GRF data, the knee rotation and moment were calculated for each participant using inverse dynamics for idealized rigid segments. All moments were normalized to body mass. Anthropometric data were calculated from individual body mass and height using Dempster's regression equations (Dempster, 1955).

Knee rotation was calculated based on the joint coordinate system definition (Grood and Suntay, 1983), which described knee rotation as occurring around the shank’s longitudinal axis. The neutral position of the knee (0° knee rotation) was defined as the knee angle five frames before foot contact (defined to occur at the first frame where vertical GRF exceeded 20 N), i.e., knee close to fully extended with no external moment affecting the limb (0 Nm knee joint moment).

For each trial (patients and controls), the maximum tibial internal rotation and corresponding net external knee joint moment about the tibia’s longitudinal axis was determined during the stance phase. Then, to avoid potential outliers, rotation and moment values from the trial with the second highest tibial internal rotation were used for further analyses. Furthermore, these values were used to calculate the rotational stiffness of each knee by dividing the change in rotational moment with the change in tibial internal rotation; the change was taken between the mentioned values and the values from the unloaded condition, defined as 0° rotation and 0 Nm moment.

### Statistical analysis

Sample size calculation was limited by the fact that in vivo rotational stiffness has not been reported in ACL deficient subjects using 3D motion analysis. Hence, measures of tibial rotation from previous studies were used in the sample size calculation (Ristanis et al., 2005, Ristanis et al., 2006). As the cohort of ACL deficient patients were to be divided into three groups afterwards, which received three different ACL reconstructions in a randomized clinical trial (RCT) (Bohn et al., 2015), the power calculation was based on the difference in tibial rotation between ACL intact and ACL reconstructed knees. According to this power calculation, a total of nine subjects were needed in each of the three groups in the RCT. To account for dropouts and problems with data retrieval from 3D motion analysis, we chose to include approximately 15 patients per group and ended up with a total of 44 ACL deficient subjects and a control group of 16 subjects.

Statistical analysis consisted of a comparison between three groups of knees: the ACLD knee, the ACLI knee and the control group (both knees). Three parameters were evaluated: internal tibial rotation, rotational moment and rotational stiffness, respectively, during each of the three tasks performed (walking, running and pivoting). The three parameters combined with the three tasks were considered as separate end points for a total of nine parameters. For each of such parameter a repeated measurements ANOVA was used with the subject ID as the repeated factor, to take into account different subjects level. The ANOVA was one way using the leg group as the factor. The residual variation, i.e. the within subject variation, was allowed to vary by group. The model was fit as a mixed model in Stata (STATA software version 14, StataCorp LP, Texas, USA). To our knowledge, it is statistically unclear which adjustment for multiple comparisons one should apply and therefore we have chosen to report the uncorrected *p*-values (Perneger, 1998). Significance level was set at *P* < 0.05.

Model validation was performed separately for each end point by inspecting (standardized) residuals, fitted values and random effect estimates (BLUP’s). Specifically, we checked distributional assumptions and for signs of heteroscedasticity. When evaluating the model we paid attention to the fact that the model was to be used for estimating mean differences and not for e.g. obtaining predictions. When initial inspections along with the model validation gave impression of a screwed distribution, the log transform was applied to the outcome and the model validation repeated. In the case of the pivot measurements, this caused us to prefer the analysis of log transformed observations.

## Results

### Knee stability and patient-related outcome scores

At the time of inclusion, none of the ACL deficient patients were graded IKDC A (normal), whereas 43 % were IKDC B (nearly normal), 39 % were IKDC C (abnormal) and 18 % were graded IKDC D (severely abnormal). The average passive, sagittal laxity measured by KT-1000 was 3.5 ± 2.2 mm.

For the ACL deficient patients, the four different patient-related outcome scores were as follows (mean ± SD): IKDC 61 ± 11, KOOS4 73 ± 13, Lysholm 72 ± 13 and Tegner 3.8 ± 1.4. The average scores for the healthy knee controls were IKDC 97 ± 4, KOOS4 97.5 ± 2.8, Lysholm 95 ± 9 and Tegner 7.5 ± 1.9.

### Three-dimensional motion analysis

During walk, both the ACLI and the ACLD knee rotated less than the knees of the control group and these differences were significant (*p* < 0.001) (Table 2). No significant difference was seen in moment or stiffness during walk between the three groups of knees, although a tendency was seen between the stiffness of the ACLD knee and the control group (*p* = 0.098).

**Table 2:**
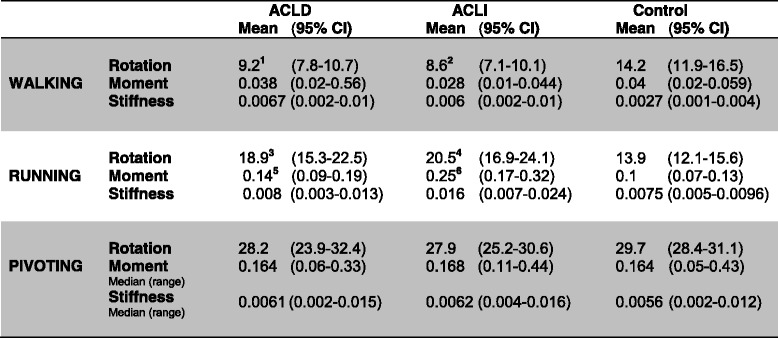
Kinematic and kinetic data from the 3D motion analysis

*ACLD* ACL deficient knee, *ACLI* contralateral ACL intact knee, Control: knee healthy control group. Rotation: tibial internal rotation, expressed in degrees (deg). Moments: net knee joint external moments, expressed as Nm/kg. Stiffness: rotational stiffness, expressed as (Nm/kg)/deg. Means and 95% Confidence intervals (CI) are reported. Pivoting/rotational moments and pivoting/stiffness were log transformed during statistical analyses; therefore, Median and range are reported for these parameters. (^1^ACLD vs. Control *p* < 0.001, ^2^ACLI vs. Control *p* < 0.001, ^3^ACLD vs. Control *p* = 0.014, ^4^ACLI vs. Control *p* = 0.001, ^5^ACLD vs. ACLI *p* = 0.015, ^6^ACLI vs. Control *p* < 0.001)

Tibial rotation during running showed a significant difference between groups. Hence, the control group displayed a significantly lower tibial rotation than both the ACLD knee (*p* = 0.014) and the ACLI knee (*p* = 0.001). Moments during running were significantly higher in the ACLI knee compared to both the ACLD knee (*p* = 0.015) and the control group (*p* < 0.001). Stiffness was not significantly different between groups, although a tendency was seen between the ACLI knee and the control group (*p* = 0.062).

Pivoting displayed no significant differences in tibial rotation, moments or stiffness between the ACLD, ACLI and control knees.

## Discussion

The most important findings of this cross-sectional study were that tibial internal rotation was not increased in ACLD knees compared to ACLI knees during any of the three tasks investigated. Thus, our findings did not support this part of our hypothesis. Furthermore, we found that the tibial internal rotation was significantly different between both knees of the ACL-injured group and the healthy control group during walking and running but not during pivoting. Furthermore, no difference in external knee joint moments was found between ACLD, ALCI and control knees during walking and pivoting. Interestingly, the ACLI knee displayed a significantly higher moment during running compared to both the ACLD knee and the control group, which could represent a compensatory strategy. Finally, rotational stiffness did not differ significantly between groups in any of the three tasks performed. Thus, none of our findings supported our hypothesis.

The pivoting maneuver applied in the present study has previously been used to investigate tibial rotation in ACLD knees (Ristanis et al., 2006, Tsarouhas et al., 2011). Similar to our study, these two studies compared ACLD knees to both the contralateral ACLI knee and a knee healthy control group. Thus, Ristanis et al., from whom we replicated our pivoting task, reported an increase in tibial rotation of the ACLD knee compared to both control groups (Ristanis et al., 2006). However, Tsarouhas et al. (Tsarouhas et al., 2011) did not find any difference in tibial rotation between ACLD, ACLI and control knees, which is in line with the findings of the current study. The pivoting maneuver performed in the latter study was, however, slightly different from the one conducted in this current study (stair descent and 60° pivoting (Tsarouhas) versus 90° pivoting (Ristanis)).

In general, higher absolute values of mean rotation during pivoting are measured in our study compared to other authors (28.5° (Table 2) vs. 15.3° (Tsarouhas) and 22.5° (Ristanis)) (Ristanis et al., 2006, Tsarouhas et al., 2011). These differences might be attributed to different definitions of neutral position (i.e., 0° knee rotation). Therefore, in our study, a neutral rotational position was defined as a knee angle five frames before foot contact, while others most often used a standing trial to define the neutral position (Ristanis et al., 2006, Tsarouhas et al., 2010, Tsarouhas et al., 2011, Webster et al., 2010). Thus, this neutral position was chosen to ensure that no external moment affected the limb, forcing zero rotation to correspond to zero moment, which makes sense mechanically because rotation is caused by moments. This furthermore provided us with two sets of corresponding moment-rotation values, enabling us to calculate rotational stiffness, defined as change in moment divided by change in rotation. However, absolute values are of lesser importance in studies where the outcome variable is a difference between absolute values (in our case, absolute ACLD and ACLI rotation angle values).

Existing literature on the kinematics of ACLD knees during walking and running is likewise inconsistent (Andriacchi and Dyrby, 2005, Takeda et al., 2014,Waite et al., 2005, Yim et al., 2015, Zabala et al., 2015). The discrepancies might be due to methodological differences, which make it difficult to compare gait analysis results across studies (Fuentes et al., 2011, Zabala et al., 2015). A possible trend towards an increased internal rotation of the tibia during walking was, however, described by Zabala et al. (Zabala et al., 2015). Although our results are not in line with the current trend, other authors have reported comparable kinematic results to ours while walking and running (Takeda et al., 2014, Yim et al., 2015). These studies did, however, compare only the ACLD knee to their contralateral ACLI knee. Similar to our results, no difference in tibial rotation during the stance phase of walking was found (Takeda et al., 2014, Yim et al., 2015). Additionally, Takeda et al. investigated tibial rotation during running and did not find any significant difference in rotation between knees, which is in line with our findings (Takeda et al., 2014). Waite et al. also investigated running in ACLD knees (Waite et al., 2005). This author reported the ACLD knee to be more internally rotated in the latter part of the stance phase compared to the contralateral uninjured knee, which was different from our findings (Waite et al., 2005).

Few studies have reported on the rotational moments in ACLD knees during natural movements (Fuentes et al., 2011, Tsarouhas et al., 2010, Tsarouhas et al., 2011). The moments during walking were described by Fuentes et al., who found a lower internal rotational moment in ACLD knees compared to a healthy control group (Fuentes et al., 2011). These findings are not in line with the current study. Only Tsarouhas et al. reported on rotational moments of force in ACLD knees during pivoting and, similar to the findings of this current study, no significant difference in rotational moments was found between knees (Tsarouhas et al., 2010, Tsarouhas et al., 2011). To our knowledge, rotational moments during running have not been reported in ACL-deficient subjects in the past. Surprisingly, we found that net external knee joint moments in both knees of the ACL-injured patients were higher than the control group during running. Additionally, our kinematic results during walking and running showed both knees of the ACL-injured group to be significantly different from our healthy control group. These findings indicate some type of adaptive or compensatory strategy for both knees of the ACL-injured patients and, furthermore, that the kinematics and kinetics of the contralateral limb are not necessarily unchanged or representative of healthy control knees when there is an ACL injury in the ipsilateral knee (Zabala et al., 2015).

As mentioned in the introduction section, the rotational stiffness in ACLD knees during natural movements has not been reported previously. Interestingly, the present study found an increase in mean rotational stiffness greater than 50 % between control knees and both knees of the ACL-injured patients during walking. These differences in means were not statistically significant, though. A possible explanation could be that the ACL-injured subjects, as a precaution to episodes of instability, activate their muscles more than healthy subjects. During running, the stiffness of both knees increases with the greater mechanical demand placed on the knee. Hence, running eliminates the double-support phase and reduces the effects of compensation from the contralateral limb. Surprisingly, the stiffness of the ACLI knee during running was more than 50 % higher than the stiffness of the ACLD knee and the control group in the current study, which we cannot explain. These differences in means were, however, not statistically significant either. Finally, we found that stiffness during pivoting was almost alike in all three groups (approximately 0.0056-0.0062 (Nm/kg)/deg), which was quite surprising. Thus, while rotational stiffness during internal rotation in all ACLI knees was provided by the ACL, passive structures and muscle contractions, the ACLD knees must be able to adapt their muscle activation to obtain a suitable rotation and, therefore, a rotational stiffness equal to the ACLI knees during pivoting. The latter statement is supported by Andriacchi et al., who stated that “adaptations to the patterns of muscle firing can compensate for kinematic changes associated with the loss of the ACL” (Andriacchi and Dyrby, 2005). Unfortunately, electromyography (EMG) measurements were not obtained in the present study; these measurements would have contributed important information on muscle activation patterns. Additionally, we did not differentiate between copers and non-copers in our study population, and this could potentially obscure genuine differences in movement patterns (Frobell et al., 2010, Rudolph et al., 2001).

In sum, an increase in internal tibial rotation and knee joint external moment was observed when a higher rotational demand was placed on the knees of all test subjects (progression from walking to running to stair descent/pivoting). However, ACLD knees did not demonstrate increased tibial rotation as hypothesized. The increased load during the different tasks was, however, not immediately reflected in the rotational stiffness, as pivoting displayed the same rotational stiffness as walking in the ACL-injured knees. Therefore, kinetic data are equally important, as the ACL is a passive, elastic structure, and the magnitude of rotation allowed by the ACL depends on the magnitude of the moment applied to by the surroundings to the leg about the tibia’s longitudinal axis. If the moment is not carefully measured and reported (similar for the compared legs), rotation is not a valid measure of the ability of the ACL to prevent rotation of the knee, i.e., provide rotational stability.

The results of the current study should be considered in light of the study’s limitations. Firstly, a cross-sectional design was used and subjects were compared at different time points since injury (Zabala et al., 2015). Because the pre-injury stiffness of both knees of the patients was not known, knee healthy subjects were selected as a control group instead. Secondly, although 3D motion analysis is widely accepted and well established for advanced functional biomechanical analysis in knee patients, numerous limitations have been described, especially the use of skin markers to predict rotational bone movements (Reinschmidt et al., 1997). To minimize this problem in the present study, marker clusters were used instead of single markers (Cappozzo et al., 1997). Cluster markers are especially useful for measuring optimized rotational measurements in 3D motion analysis. Furthermore, it has been shown that during simultaneously measured knee motion using an optical tracking system and dynamic radiostereometric analysis (RSA), internal/external rotation was fairly similar up to 25° of flexion (Tranberg et al., 2011). As shown in Fig. 1, the pivoting task in this study was performed on an almost extended leg. Thirdly, one of the pairs of corresponding moment-rotation values used for stiffness calculation was 0°, 0 Nm; we defined 0° rotation as the knee rotation angle just prior to ground contact, and assumed the corresponding knee joint moment to be 0, because the moment created by the GRF at this instance by definition was 0; however, while no GRF moment present implies that no counteracting knee joint moment is necessary, knee muscle activity might still have created a knee joint moment and affected the rotation angle. Finally, our cohort consisted of both females and males, which might increase the variability in our data, as several static studies have shown female knees to be more lax and less stiff than male knees (Hsu et al., 2006, Shultz et al., 2012).

## Conclusions

In conclusion, we found that a high-intensity activity combining stair descent and pivoting produces similar tibial internal rotations, net knee joint external moments and rotational stiffness in ACL deficient knees compared to contralateral ACL intact knees and a knee healthy control group. During running, the ACL intact knees displayed a higher external moment than the ACL deficient knees and the knee healthy control group. This could indicate muscular adaption or a protective strategy in the ACL-injured patients.
